# Production of the Anti-Inflammatory Compound 6-*O*-Palmitoyl-3-*O*-β-D-glucopyranosylcampesterol by Callus Cultures of *Lopezia racemosa* Cav*.* (Onagraceae)

**DOI:** 10.3390/molecules19068679

**Published:** 2014-06-24

**Authors:** Roberta Salinas, Jesús Arellano-García, Irene Perea-Arango, Laura Álvarez, María Luisa Garduño-Ramírez, Silvia Marquina, Alejandro Zamilpa, Patricia Castillo-España

**Affiliations:** 1 Centro de Investigación en Biotecnología, Universidad Autónoma del Estado de Morelos, Av. Universidad 1001, Cuernavaca CP 62209, Morelos, Mexico; E-Mails: robertsm76@gmail.com (R.S.); jesus.arellano@uaem.mx (J.A.-G.); iperea@uaem.mx (I.P.-A.); 2 Centro de Investigaciones Químicas, Universidad Autónoma del Estado de Morelos, Av. Universidad 1001, Cuernavaca CP 62209, Morelos, Mexico; E-Mails: lalvarez@uaem.mx (L.Á.); lgarduno@uaem.mx (M.L.G.-R.); smarquina21@hotmail.com (S.M.); 3 Centro de Investigación Biomédica del Sur, Instituto Mexicano del Seguro Social, Argentina No. 1, Xochitepec CP 62790, Morelos, Mexico; E-Mail: azamilpa_2000@yahoo.com.mx

**Keywords:** *Lopezia racemoza* Cav., calli, anti-inflammatory activity, TPA, cytotoxic activity

## Abstract

*Lopezia racemosa* Cav. is a plant used in Mexican traditional medicine to heal inflammatory diseases. From this plant we isolated the novel compound 6-*O*-palmitoyl-3-*O*-β-D-glucopyranosylcampesterol (**1**) and 6-*O*-palmitoyl-3-*O*-β-D-glucopyranosyl-β-sitosterol (**2**), previously reported to have cytotoxic activity on several cancer cell lines. We evaluated the anti-inflammatory activity of **1** *in vivo* by mouse ear edema induced with 12-*O*-tetradecanoylphorbol-13-acetate (TPA) and 57.14% inhibition was observed. The aim of our study was to obtain callus cultures derived from this plant species with the ability to produce the compounds of interest. Callus cultures were initiated on MS basal medium amended with variable amounts of naphthaleneacetic acid (NAA), or 2,4-dichlorophenoxyacetic acid (2,4-D), combined or not with 6-benzylaminopurine (BAP). Ten treatments with these growth regulators were carried out, using *in vitro* germinated seedlings as source of three different explants: hypocotyl, stem node, and leaf. Highest yield of **1** was observed on callus derived from leaf explants growing in medium containing 1.0 mg/L 2,4-D and 0.5 mg/L BAP. Selected callus lines produced less **1** than wild plants but the *in vitro* cultured seedlings showed higher production. So we conclude that it could be attractive to further investigate their metabolic potential.

## 1. Introduction

Plants are an important source of new products with medicinal value for drug development. Nowadays several chemicals derived from plants are drugs currently used in many countries around the world [1]. The search for new plant-derived chemicals should be a priority in current and future efforts toward sustainable conservation and rational utilization of biodiversity [2]. There are some biotechnological approaches to produce large amounts of desirable medicinal compounds from plants, particularly, plant tissue culture have potential as an alternative to traditional agriculture for industrial production of bioactive plant metabolites [3]. Some strategies using *in vitro* systems had been extensively studied to improve the production of plant chemicals with anticancer activity such as taxol, docetaxel, camptothecin, podophyllotoxin, vincristin and vinblastine. These substances have complex chemical structures and despite advances in synthetic chemistry its production still depends directly or indirectly on semi-synthesis from biological sources [4].

According to the World Health Organization 2012 report, cancer was the cause of 8.2 million deaths worldwide. Increase in world population is raising the demand for medicines and new drugs to cope with cancer and other diseases [5,6]. Since inflammation is a suffering closely linked to diseases like cancer, finding of anti-inflammatory drugs could be useful to discover new drugs with anticancer activity. Plants are an alternative source of new active principles to treat symptoms of chronic inflammation associated or not with benign or malignant neoplasm. *Lopezia recemosa* commonly known as punch herb or cancer herb is a plant used in Mexican traditional medicine to heal inflammatory diseases [7]. A recent report showed that some fractioned extracts of this plant possess antimicrobial, antiparasitic, anti-inflammatory and cytotoxic activities [8].

**Figure 1:**
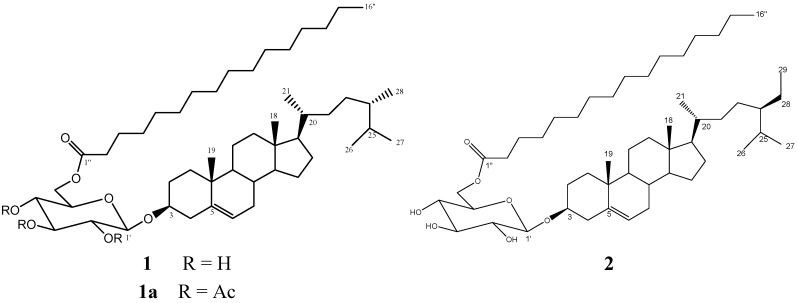
Chemical structure of compounds **1** and **2**.

In this paper we report the bioassay-guided chromatographic purification of the novel anti-inflammatory compound 6-*O*-palmitoyl-3-*O*-β-D-glucopyranosylcampesterol (**1**), together with the known 6-*O*-palmitoyl-3-*O*-β-D-glucopyranosyl-β-sitosterol (**2**) (Figure 1). Also, the *in vitro* cultured seedlings and calli cultures derived from different tissues of *L. racemosa*, able to produce **1**, are described.

## 2. Results and Discussion

### 2.1. Bioassay-Guided Chromatographic Purification and Characterization of Compound **1**

In a bioassay-guided study carried out to evaluate some previously obtained anti-inflammatory activity of *L. racemosa* extracts, the hexane (SMH), dichloromethane (SMD) and methanol (SMM) extracts were tested by the *in vivo* model of mouse ear inflammation induced with TPA as reported by Qadeer, G *et al.* [9]. Our results showed that the extract with higher anti-inflammatory activity was SMD, with 58.3% of inhibition (Table 1). Then the SMD extract was fractionated by column chromatography obtaining one active fraction (LR50B) which displayed 86.26% inhibition of the inflammation. Later on this fraction was subjected to rechromatography and our novel compound **1** was obtained mixed with 6-*O*-palmitoyl-3-*O*-β-D-glucopyranosyl-β-sitosterol (**2**). This acyl glucosyl sterol mixture was obtained with a ratio of 23:1, respectively. Finally, the isolation of **1** was achieved by precipitating with acetone and recrystallizing, and it was characterized as its triacetyl derivative **1a**. Compound **2** was obtained in pure form by HPLC purification and was identified on the basis of the comparison of its spectroscopic data with those of the described. This compound has cytotoxic activity on several cancer cell lines such as breast, prostate, T-cell leukemia and Burkitt lymphoma [10].

**Table 1:** Anti-inflammatory activity of the extracts, fractions LR50 A-H and compound **1**.

Sample	Percentage of Anti-Inflammatory Activity (1 mg/ear)	IC_50_ (mg/ear)
SMM *^a^*	16.81 ± 0.96	>1
SMD *^b^*	58.40 ± 1.63	0.93 ± 0.06
SMH *^c^*	48.74 ± 1.01	>1
LR50A	35.30 ± 1.44	>1
LR50B	86.26 ± 1.89	ND *^e^*
LR50C	−30.72 ± 1.49	>1
LR50D	19.47 ± 2.21	>1
LR50F	11.26 ± 2.57	>1
LR50G	33.78 ± 2.54	>1
LR50H	31.30 ± 1.48	>1
1 *^d^*	57.14 ± 1.81	0.45 ± 0.08
INDOMETHACIN	91.35 ± 0.87	0.14 ± 0.09

For all samples acetone was used as vehicle; *^a^* Methanolic extract; *^b^* Dichloromethane extract; *^c^* Hexanoic extract; *^d^* 6-*O*-palmitoyl-3-*O*-β-d-glucopyranosylcampesterol; *^e^* Not determinated.

Compound **1a** gave a pseudomolecular ion [M+Na]^+^ peak at *m/z* 949.6738 by HRESIMS, indicating the molecular formula C_56_H_94_O_10_Na. NMR spectral data showed the characteristic signals of campesterol [δ 0.60 (3H, s, H-18), 0.91 (3H, s, H-19), 0.74 (6H, d, *J* = 7.2 Hz, H-25, H-26), , 0.72 (3H, d, *J* = 6.5 Hz, H-28), 0.84 (3H, d, *J* = 6.4 Hz, H-21) ] with a hexose unit [99.7 (C-1'), 71.6 (C-2'), 73.0 (C-3'), 68.8 (C-4'), 71.8 (C-5'), and 62.1 (C-6')] and a long aliphatic chain [11]. The ^13^C-NMR spectrum further indicated the presence of an ester carbonyl at δ_C_ 173.6, in addition to the three carbonyl carbons from the acetates at 169.5, 169.56, and 170.5, as well as a long aliphatic chain (δ 34.2, 29.8, 24.9, and 14.28). The ^1^H- and ^13^C-NMR data of **1a** were almost identical to those of compound **2** except that **1a** contained a C-28 steroidal system ring of campesterol instead of β-sitosterol on compound **2**, which was supported by the ion peak at *m/z* 400.6 corresponding to the aglycon, and at *m/z* 239 corresponding to the palmitoyl moiety, in the mass spectrum. Basic hydrolysis of the natural product **1** afforded palmitic acid, identified by GC-MS analysis, while acid hydrolysis yielded campesterol, identified by comparison with an authentic sample. HMBC correlations from H-1' (δ 4.05) → C-2' (δ 71.6) and C-3 (δ 80.2) indicated that the sugar residue was attached at C-3 of the campesterol aglycon. The aliphatic chain is attached at C-6’ position of the glucose unit through an ester linkage. This fact was confirmed by the long range HMBC correlations observed between the carbonyl carbon at δ 173.6 of the ester moiety and the diasterotopic protons H-6a and H-6b of the sugar unit at δ_H_ 4.15 (1H, dd, *J* = 5.2, 12.0 Hz) and 4.05 (1H, dd, *J* = 2.4, 12.0 Hz). On the basis of the above results the novel compound **1** was characterized as 6-*O*-palmitoyl-3-*O*-β-D-glucopyranosyl-campesterol.

Compound **1** was evaluated by the same *in vivo* model of mouse ear edema induced by TPA and an inhibition of 57.14% was observed as compared with indomethacin (91.35%). The IC_50_ values of the anti-inflammatory activity were calculated using the GraphPad Prism^®^ software; finding that the values for compound **1** and indomethacin were 0.45 ± 0.08 and 0.14 ± 0.09, respectively (Table 1). The anti-inflammatory activity difference between the fraction LR50B and isolated compound **1** could be explained by a synergistic effect of both compounds in the mixture.

Due that the fact compound **1** is obtained in very low yield, and with the aim of obtaining different lines of calli cultures of *L. racemosa* with the ability to produce the anti-inflammatory compound **1** at concentrations higher or equal to those found in wild plants, but grown under controlled and reproducible laboratory conditions, we undertook the production of different lines of calli cultures of *L. racemosa* using different tissue culture techniques.

### 2.2. Callus Production

Callus formation started after one week on treatments containing any combination of NAA/BAP or 2,4-D/BAP in MS [12] culture medium. No response was observed to treatments lacking growth regulators or the one containing BAP as the sole growth regulator, both in photoperiod or darkness. Out of the forty eight culture combinations of treatments containing 2,4-D or NAA with or without 0.5 mg/L BAP, different explants and incubation conditions, only one did not produce callus during the first week, while forty seven showed some callus development. However, after thirty days on callus induction media, eleven of such combinations, mainly those explants grown on NAA/BAP, showed morphogenetic responses, while all of those exposed to 2,4-D/BAP remained undifferentiated (Table 2). Calli derived from each one of all these combinations were denominated lines. Each line was named according with the origin of its explant, the nature of the plant growth regulator used and its concentration, and their incubation condition.

**Table 2:** Callus formation response in different combinations of plant growth regulators, explants, and incubation conditions that resulted in thirty seven different lines of *Lopezia racemosa* Cav. calli after thirty days of culture.

Treatments (mg/L)	Photoperiod			Darkness		
	Leaf	Stem Node	Hypocotyl	Leaf	Stem Node	Hypocotyl
1) NAA 0.5	**L *^b^***	**S *^b^***	**H *^b^***	**L *^b^***	**S *^ad^***	**H *^b^***
2) NAA 1.0/BAP 0.5	**L *^a^***	**S *^a^***	**H *^a^***	**L *^a^***	**S *^a^***	**H *^a^***
3) NAA 2.0/BAP 0.5	**L *^b^***	**S *^b^***	**H *^a^***	**L *^a^***	**S *^b^***	**H *^ad^***
4) NAA 4.0/BAP 0.5	**L *^b^***	**S *^b^***	**H *^a^***	**L *^a^***	**S *^b^***	**H *^a^***
5) 2,4-D 0.5	**L *^a^***	**S *^ad^***	**H *^a^***	**L *^a^***	**S *^ad^***	**H *^a^***
6) 2,4-D 1.0/BAP 0.5	**L *^ad^***	**S *^a^***	**H *^a^***	**L *^a^***	**S *^a^***	**H *^a^***
7) 2,4-D 2.0/BAP 0.5	**L *^a^***	**S *^a^***	**H *^a^***	**L *^a^***	**S *^a^***	**H *^a^***
8) 2,4-D 4.0/BAP 0.5	**L *^a^***	**S *^a^***	**H *^a^***	**L *^a^***	**S *^a^***	**H *^a^***
9) BAP 0.5	**L *^c^***	**S *^c^***	**H *^c^***	**L *^c^***	**S *^c^***	**H *^c^***
10) Without PGR	**L *^c^***	**S *^c^***	**H *^c^***	**L *^c^***	**S *^c^***	**H *^c^***

***^a^*** Explants that only produced calli; ***^b^*** Explants with morphogenetic calli; ***^c^*** Explants that did not respond; ***^d^*** Selected calli.

### 2.3. Callus Lines Selection

Selection of calli was carried out by the phenotypic characteristics observed during subsequent subcultures of the thirty seven lines obtained during the previous step. Lines showing oxidation processes were discarded and lines which showed stable growth and friability appearance were sub-cultivated during three months. Finally, only five lines were chosen based on their friability and growth rate (Table 3): (1) SN0.5K; (2) HN2.0B0.5K; (3) SD0.5P; (4) SD0.5K and (5) LD1.0B0.5P. Lines LD1.0B0.5P and SD0.5K, showed the faster process of callus induction, growth rate and preserved the callus phenotype with more stability than others lines selected. All five lines growth faster after the second month and produce enough biomass for compound **1** evaluation.

**Table 3:** Quantify of compound **1**, in callus selected lines, wild plants and seedlings of *Lopezia racemosa* Cav.

Callus Line or Source of 1	Biomass DW(g)	DCM Extract (mg)	µg of 1 in DCM Extract ± SD	µg of 1/g Biomass ± SD
SN0.5K	2.25	22.55	38.61 ± 3.16	17.17 ± 1.38
HN2.0/B0.5K	3.34	21.45	153.21 ± 5.13	45.80 ± 1.53
SD0.5P	2.61	37.41	289.27 ± 50.64	110.83 ± 19.40
SD0.5K	3.07	36.85	493.09 ± 14.26	160.61 ± 4.61
LD1.0/B0.5P	3.91	73.15	678.54 ± 90.08	173.98 ± 23.09
Wild Plants	3170.01	31,304.01	4,935,000.60 ± 3377.22	1556.80 ± 103.92
Seedlings	10.81	124.21	23,070.60 ± 1292.98	2130.13 ± 120.02

S = Stem node; H = Hypocotyl; L = Leaf; N = NAA; D = 2,4-D; B = BAP; K = Darkness; P = Photoperiod.

### 2.4. Compound **1** Production

**Figure 2:**
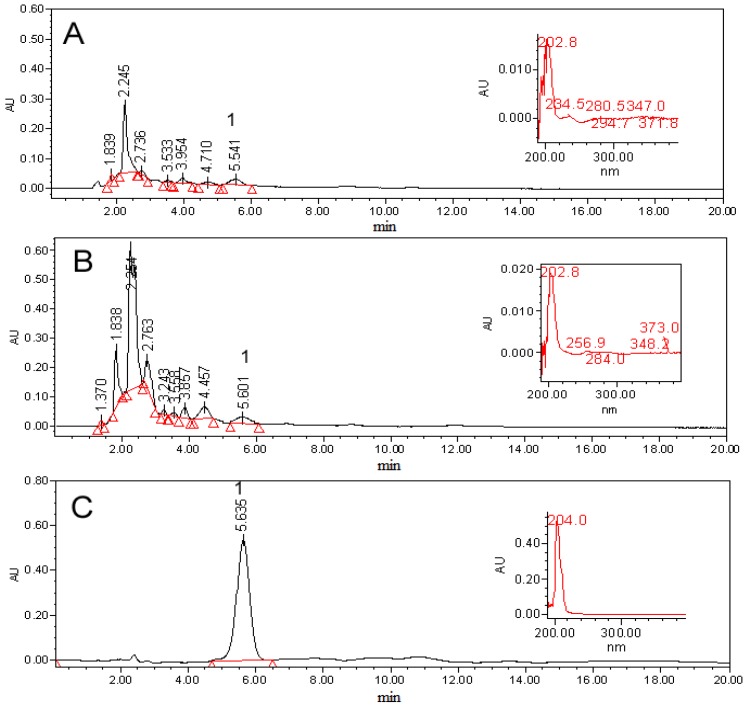
HPLC patterns showing the presence of compound **1** in: (**A**) Wild plant, and (**B**) *In vitro* culture seedlings, in comparison with (**C**) Compound **1** isolated from wild plant of *L. racemosa*.

A calibration curve for **1** was generated from purified samples and it was used to quantify the DCM extracts from the five calli lines selected and established. The callus line with greater production of biomass (3.91 g) and **1** (174.0 µg/g of biomass) was LD1.0B0.5P (Table 3). However, all five callus line cultures selected produced **1**, despite the fact they were cultivated under different illumination conditions, which indicate that **1** is produced in both conditions photoperiod and continuous darkness, regardless of the explants and the plant growth regulator concentration. However, it is clear that all three calli selected lines growing in the presence of 2,4-D, grew faster and produced more **1** than those growing in the presence of NAA. From our results it is clear that this plant species has the potential to produce **1** and that this compound is produced in all evaluated calli lines, but they produce significantly less **1** than wild plants. However, the fact that the *in vitro* culture seedlings showed higher production than wild plants (Figure 2), could be attractive to investigate the *in vitro* metabolic capacity of different tissues of this plant species to produce compound **1**.

*Conditions*: Lichrospher Si 60 (258 × 5 mm Merck) column, mobile phase: propane-2-ol:*n*-hexane (20:80 v/v); flow rate, 1.0 mL/min^−1^. A long wave of 205 nm was used to detect compound **1**.

## 3. Experimental

### 3.1. Chemical Experimental Procedures

Optical rotations were measured in CH_3_OH on a Perkin-Elmer 241 digital polarimeter at 25 °C. IR data were recorded using a Vector 22 Bruker spectrophotometer. All NMR spectra were acquired with a Varian–Unit 400 at 400 MHZ in CDCl_3_. Chemical shift are reported in parts per million (ppm) relative to tetramethylsilane (TMS) as reference. EIMS and HRESIMS data were obtained using a JEOL JMS-AX 505 HA instrument equipped with electrospray ionization (ESI) source. All MS experiments were performed in positive ion mode. HPLC analysis were performed on a Waters Delta prep 4000 instrument (Water Co., Milford, MA, USA) equipped with a U6K injector, 606 E pump, Millenium 3.2 software and 966 diode array detector.

### 3.2. Plant Material

Plants and mature fruits were collected in February 2010 from 30 km of the Mexico-Cuernavaca federal road in Morelos, Mexico. A voucher specimen has been deposited at HUMO-Herbarium of the Biodiversity and Conservation Research Center of the Autonomous University of Morelos State at Cuernavaca, Morelos, Mexico. The plant material was identified by Gabriel Flores Franco of HUMO-Herbarium and was registered with the code number 22029. Seeds were obtained from screening fruits and were preserved at 4 °C.

### 3.3. Extraction and Isolation of **1** from Wild Plant

Whole foliage dry plant material (2.5 kg) was extracted sequentially with hexane, dichloromethane (CH_2_Cl_2_) and methanol. The dichloromethane fraction was evaporated to dryness to afford the crude extract (21 g), which was fractionated by 70–230 mesh silica gel using CH_2_Cl_2_-acetone−CH_2_Cl_2_−MeOH gradient elution. The fraction eluted with 80:20 CH_2_Cl_2_–acetone (1.8 g) was separated by column chromatography (CC) eluting with CH_2_Cl_2_-acetone, to afford six fractions: LR50A, 0.0370 g (100:0)), LR50B 0.0703 g (99:1, 98.5:1.5, 97:03), LR50C, 0.0638 g (96:04), LR50D, 0.1010 g (95:05), LR50E, 0.0854 g (90:10), LR50F, 0.2520 g (80:20), LR50G, 0.0420 g (70:30), LR50H, 0.1243 g (60:40). Fraction LR50B (70.3 mg) was separated by column chromatography (CC) eluting with hexane–ethyl acetate. Fractions eluting with 96:4 hexane–ethyl acetate contained a mixture of two compounds, an aliquot of this fraction was re-crystallized from acetone to give 7 mg of compound **1**, and 5 mg of compound **2**. Compound **2** was further purified by HPLC on a Lichrospher Si 60 (258 × 5 mm Merck) column with propane-2-ol:*n*-hexane, 20:80 v/v as isocratic eluent system and a flow rate of 1.0 mL min^−1^, to afford 2 mg of compound **2** (Tr = 4.7 min). The other part of the fraction was acetylated with acetic anhydride (2 mL) and pyridine (1 mL), the residue was quenched with 3 mL of water and extracted with dichloromethane, Si-gel chromatographic purification, using a isocratic mixture of *n*-hexane/EtOAc (95:5) afforded 25 mg of **1a** [10]. 

### 3.4. Compound **1a**

Colorless waxy solid (CHCl_3_); mp 118–120 °C; 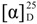 +0.076 (c 0.1, CHCl_3_); IR (Nujol) *ν*_max_ 1756.8 cm^−1^; ^1^H-NMR (CDCl_3_, 400 MHz): δ_H_ 5.28 (1H, d, *J* = 4.8 Hz, H-6), 5.13 (1H, t, *J* = 10.0 Hz, H-3'), 4.05 (1H, d, *J* = 7.6 Hz, H-1'), 4.97 (1H, t, *J* = 9.2 Hz, H-4'), 4.88, (1H, t, *J* = 9.6 Hz, H-2'), 4.15 (1H, dd, *J* = 5.2, 12.0 Hz, H-6'a), 4.05 (1H, dd, *J* = 2.1, 12.0 Hz, H-6'b), 3.61 (1H, ddd, *J* = 2.4, 5.6, 10.0 Hz, H-5'), 3.41 (1H, m, H-3), 2.25 (2H, m, H-2''), 2.16 (2H, m, H-4), 1.98 (3H, s, CH_3_CO), 1.95 (3H, s, CH3CO), 1.93 (3H, s, CH_3_CO), 1.94 (2H, m, H-12), 1.8 (2H, m, H-2), 1.60 (1H, m, H-25), 1.58 (2H, m, H-3''), 1.57 (2H, m, H-15), 1.44 (2H, m, H-11), 1.40 (1H, m, H-8), 1.33 (1H, m, H-20), 1.26 (2H, m, H-22), 1.25 (24H, m, aliphatic chain), 1.2 (2H, m, H-7), 1.18 (2H, m, H-28), 1.16 (2H, m, H-16), 1.06 (1H, m, H-17), 0.98 (2H, m, H-1), 0.94 (1H, m, H-14), 0.91 (3H, s, H-19), 0.86 (1H, m, H-24), 0.84 (3H, d, *J* = 6.4 Hz, H-9, H-21), 0.80 (3H, t, *J* = 6.8 Hz, H-29), 0.77 (3H, t, *J* = 6.0 Hz, H-16''), 0.75 (6H, s, H-26, H-27), 0.60 (3H, m, H-18); ^13^C NMR (CDCl3, 100 MHz): δ_C_ 173.6 (C-1''), 169.5 (COCH_3_), 169.56 (COCH_3_), 170.5 (COCH_3_), 140.4 (C-5), 122.3 (C-6), 99.7 (C-1'), 80.2 (C-3), 73.0 (C-3'), 71.8 (C-5'), 71.6 (C-2'), 68.8 (C-4'), 62.1 (C-6'), 56.8 (C-14), 56.2 (C-17), 50.2 (C-9), 45.9 (C-24), 42.4 (C-13), 39.8 (C-12), 39.0 (C-4), 37.3 (C-1), 36.8 (C-10), 36.2 (C-20), 34.3 (C-2''), 34.0 (C-22), 32.1 (C-7), 32.0 (C-8), 29.2 (C-25), 28.3 (C-2), 26.1 (C-16), 24.9 (C-3''), 24.4 (C-15), 21.1 (C-11), 20.89 (CH_3_CO), 20.82 (CH_3_CO), 20.7 (CH_3_CO), 19.9 (C-27), 19.4 (C-26), 19.1 (C-19), 18.9 (C-21), 14.3 (C-16''), 12.2 (C-28), 11.9 (C-18) ; HRESIMS *m/z* 949.6738 (calcd for C_56_H_94_O_10_Na, 949.6744).

### 3.5. Compound **2**

Colorless amorphous powder; IR (Nujol) *v*_max_ 1748.2 cm^−1^; ^1^H-NMR (CDCl_3_, 400 MHz): δ_H_ 5.21 (1H, m, H-6), 4.12 (1H, d, *J* = 7.5 Hz, H-1'), 4.07 (1H, m, H-2'), 3.59 (1H, m, H-3), 3.43 (1H, m, H-3'), 3.36 (1H, m, H-6'a), 3.33 (1H, m, H-6'b), 3.30 (1H, m, H-4'), 3.21 (1H, ddd, *J* = 2.0, 6.4, 8.8 Hz, H-5'), 2.25 (2H, m, H-2''), 1.18 (2H, m, H-28), 0.89 (3H, s, H-19), 0.82 (3H, d, *J* = 7.0 Hz, H-21), 0.77 (3H, t, *J* = 6.5 Hz, H-16''), 0.73 (6H, d, *J* = 6.8 Hz, H-26, H-27), 0.70 (3H, m, H-18); ^13^C NMR (CDCl_3_, 100 MHz): δ_C_ 174.3 (C-1''), 140.4 (C-5), 122.2 (C-6), 101.4 (C-1'), 79.9 (C-3), 76.3 (C-3'), 73.5 (C-5'), 70.6 (C-2'), 68.3 (C-4'), 63.8 (C-6'), 57.0 (C-14), 56.4 (C-17), 50.3 (C-9), 46.0 (C-24), 42.5 (C-13), 40.0 (C-12), 39.1 (C-4), 37.5 (C-1), 36.9 (C-10), 36.4 (C-20), 34.5 (C-2''), 34.2 (C-22), 32.2 (C-7), 32.1 (C-8), 29.4 (C-25), 28.5 (C-2), 26.4 (C-16), 25.2 (C-3''), 24.5 (C-15), 22.8 (C-28), 21.3 (C-11), 20.0 (C-27), 19.6 (C-26), 19.3 (C-19), 19.0 (C-21), 14.4 (C-16''), 12.2 (C-29). These data match those in the literature [10].

### 3.6. Acid Hydrolysis of Compound **1**

Compound **1** (2 mg) was heated with aqueous 2 mol/L HCl–1,4-dioxane (1:1, 2 mL) at 80 °C for 6 h. The aglycon was extracted with chloroform, and analyzed by GC/MS using a sample of campesterol as standard.

### 3.7. Seed Germination and Obtaining Axenic Seedling

Prior to *in vitro* culture, mature seeds were surface sterilized in smalls bags of filter paper dipped in 70% (v/v) ethanol for 1 min, followed by 15% (v/v) domestic bleach solution (6% active chlorine) under constant agitation for 15 min, then rinsed four times with sterile distilled water. All this process was carried out in laminar flow hood. Sterile seeds were transferred to baby food jars containing half-strength MS [12] medium salts and organic components, 1.5% (w/v) of sucrose, 0.1% (w/v) activated charcoal, 0.4% (w/v) Phytagel, without plant growth regulators, pH adjusted to 5.6 ± 0.1, previous to autoclaving at 108 kPa and 121 °C for 20 min. To determine the best conditions for germination and seedling development, the cultures were incubated on two different light conditions: 16/8 h (light/dark) photoperiod under white light (27 mmol m^2^ s^−1^) at 24 °C, or continuous darkness (baby jars wrapped with aluminium foil) at 24 °C. Three replicates containing ten seeds for each condition were carried out. Monitoring of seed germination was done using stereoscopic microscopy (Motic Digital Microscope DM 143, Richmond, British Columbia, Canada).

### 3.8. Callus Induction

Thirty day old axenic seedlings growing in photoperiod were used as the source of the three different explants used: leaf, hypocotyl and stem node. Each type of explant was cut around 10 mm long. The culture medium used to perform the process of callus induction consisted of full-strength MS, 3% (w/v) sucrose, 100 mg L^−1^ *myo*-inositol, pH 5.6 ± 0.1, 0.4% (w/v) Phytagel, and supplemented with variable concentrations of NAA or 2,4-D (0, 0.5, 1.0, 2.0, 4.0 mg L^−1^) without or in combination with BAP (0.5 mg L^−1^), to determine the best auxin-cytokinin combination and concentration, and the best explant type for callus induction. Each treatment consisted of five baby food jars, and three explants of each type per flask. This step was performed and maintained for 30 days in two environmental conditions: photoperiod 16/8 h (light/darkness) provided by cold fluorescent lamps (54 mmol m^2^ s^−1^) at 24 ± 1 °C and continuous darkness at 24 ± 1 °C. The effect of different auxin/cytokinin combinations on callus production was evaluated based on the appearance, the consistency, and the fresh weight of the produced calli, in each treatment (data not shown).

### 3.9. Calli Selection

From the established cultures, five lines were selected according with their displayed phenotype and their growth rate. The selected lines were: SN0.5K (0.5 μg L^−1^ NAA), HN2.0B0.5K (2.0 NAA/0.5 μg L^−1^ BAP), SD0.5P (0.5 μg L^−1^ 2,4-D), SD0.5K (0.5 μg L^−1^ 2, 4-D) and LD1.0B0.5P (1.0 μg L^−1^ 2,4-D/0.5 μg L^−1^ BAP). Callus tissue was subcultured monthly for 60 days in the same conditions before described.

### 3.10. Extraction of **1** from Selected Callus Lines

Calli were harvested and appropriately dried several times during culture to have enough plant material from each selected line. Then the dried callus material derived from each selected line was pulverized manually and separately subjected to two successive macerations with 200 mL hexane and with 200 mL of dichloromethane at room temperature. Afterwards the dichloromethane extracts were evaporated to dryness under vacuum at 38 °C in a rotaevaporator (Buchi R-200, Flawil, Switzerland), reconstituted in an HPLC solvent system of propane-2-ol:*n*-hexane (20:80 v/v), filtered through acrodiscs (pore size 0.4 mm), and applied to an HPLC column.

### 3.11. Identification and Quantification of Compound **1** in Selected Calli Lines

Thin layer chromatography for identification of **1** was done on silica gel using dichloromethane–acetone (70:30) as the mobile phase. Spots were visualized by ammonium ceric sulphate. High pressure liquid chromatography was performed using a Waters Delta prep 4000 chromatograph equipped with a Waters 717 plus Autosampler and 996 photodiode array, and refractive index detectors (Waters Co., Milford, MA), and a Lichrosfer Si 60 258 × 4 mm, and a 5 mm (Merck, Darmstadt, Germany) column. The analysis was run under isocratic conditions (propane-2-ol:*n*-hexane, 20:80 v/v) at a flow rate of 1.0 mL/min, and using 20 µL sample injections. Suitable calibration curve for quantification of the *in vitro* compounds were constructed using the active product of the wild plant as a standard. The compound was dissolved in the mobile phase at concentrations of 35.75, 71.50, 143.00, 286.00 and 572.00 µg mL^−1^ and had a retention time of 5.6 min (λ = 205 nm). A fourfold concentration of analyte solution was injected in three replicates; the calibration curve was constructed by plotting the peak areas *versus* the concentration of **1**, to yield an equation y = 2,418.2x + 49,039 (r^2^ = 0.9978). The calibration curves were based on the peak areas of the HPLC chromatograms. Retention time peaks of compound **1** from *in vitro* culture seedlings and all calli cultures were compared with that of the authentic **1** isolated from wild plant. Values were expressed as percentages on the basis of µg of **1**/biomass dry weight in grams.

### 3.12. Model of Mice Ear Inflammation Induced with TPA

Mouse ear edema was evaluated following the described protocol. All experiments were carried out using six animals per treatment. Adult male CD-1 mice with a body weight ranging from 25–30 g were used. Experiments were performed according to the Official Mexican Rule: NOM-062-ZOO-1999 Guidelines (Technical Specifications for the Production, Care, and Use of Laboratory Animals) and international ethical guidelines for the care and use of experimental animals. Mice were maintained under standard laboratory conditions (Bioterio-UAEM) at 22 °C ± 3 °C, 70% ± 5% of humidity, 12 h light/dark cycle and food/water *ad libitum*.

A negative control group received acetone as vehicle and indomethacin was used as anti-inflammatory drug as positive control group. Finally, the extracts, fractions and compounds were tested by separate treatment groups.

Animal ear inflammation was induced with 2.5 µg of TPA dissolved in 20 µL of acetone applied to the internal and external surface of the right ear to cause edema. Sample doses of 1 mg/ear of the extracts, fractions, and compound **1**, as well as the anti-inflammatory drug of reference (indomethacin) were applied. All the samples of the different treatments were dissolved in acetone and applied topically on the right ear immediately after TPA application; on the left ear acetone was applied as vehicle. Four hours after application of the samples of interest as possible anti-inflammatory agents, the animals of each treatment were sacrificed by cervical dislocation. Circular sections of 6 mm in diameter were taken from both: the treated (t) and the non-treated (nt) ears, which were weighed to determine the inflammation. Percentage of inhibition was determined by the formula expressed below:

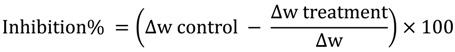
(1)
where ∆w = w_t_ − w_nt_; being w_t_ the weight of the section of the treated ear and w_nt_ the weight of the section of the non-treated ear. The IC_50_ values of the anti-inflammatory activity were calculated using GraphPad Prism^®^ software.

## 4. Conclusions

This work demonstrate that it is possible to obtain calli cultures and selected calli lines derived from different tissues of *L. racemosa* Cav, that produce the compound **1**, which is a novel compound that possesses anti-inflammatory activity. Furthermore, we found that *in vitro* cultured seedlings are able to produce higher amounts of **1** than wild plants. There are several strategies to increase the production of secondary metabolites which are produced by tissue culture techniques. However the first step is to obtain plant cell lines that have the ability to produce the compound of interest. Based on our achievements, it could be interesting to test elicitors to increase the production of **1** and other strategies such as the massive production of plantlets with the ability to produce this compound or obtaining hairy roots by genetic transformation.

## References

[1] Mulabagal V., Hsin-Sheng T. (2004). Plant Cell Cultures: An Alternative and Efficient Source for the Production of Biologically Important Secondary Metabolites. Int. J. App. Sci. Eng..

[2] Karuppusamy S. (2009). A review on trends in production of secondary metabolites from higher plants by *in vitro* tissue, organ and cell cultures. J. Med. Plants Res..

[3] Rao S.R., Ravishankar G.A. (2002). Plant cell cultures: Chemical factories of secondary metabolites. Biotechnol. Adv..

[4] Oksman-Caldentey K.M., Inze D. (2004). Plant cell factories in the post-genomic era: New ways to produce designer secondary metabolites. Trends Plant. Sci..

[5] Bhanot A., Sharma R., Noolvi M.N. (2011). Natural sources as potential anti-cancer agents: A review. Inter. J. Phytomed..

[6] Rodzi R., Cheah Y.L., Ooi1 K.K., Othman F., Mohtarrudin N., Tohid S.F., Suhaili Z., Zakaria Z.A. (2013). Chemopreventive potential of methanol extract of *Dicranopteris linearis* leaf on DMBA/croton oil-induced mouse skin carcinogenesis. Afr. J. Pharm. Pharmacol..

[7] Alonso-Castro A.J., Villarreal M.L., Salazar-Olivo L.A., Gomez-Sanchez M., Dominguez F., Garcia-Carranca A. (2011). Mexican medicinal plants used for cancer treatment: Pharmacological, phytochemical and ethnobotanical studies. J. Ethnopharm..

[8] Cruz Paredes C., Bolívar Balbás P., Gómez-Velasco A., Hernández L.R., Juárez Z.N., Sánchez Arreola E., Bach H. (2013). Antimicrobial, Antiparasitic, Anti-Inflammatory, and Cytotoxic Activities of *Lopezia racemosa*. Sci. World J..

[9] Qadeer G., Rama N.H, Hill R.A, Garduño-Ramírez M.L. (2007). Synthesis and anti-inflammatory activity of fluorinated isocoumarins and 3,4 dihydroisocoumarins. J. Fluorine Chem..

[10] Rubnov S., Kashman Y., Rabinowitz R., Schlesinger M. (2001). Suppresors of Cancer Cell Proliferation from Fig (*Ficus. carica*) Resin: Isolation and structure elucidation. J. Nat. Prod..

[11] Frenkel M., Marsh K.N. (1994). Spectral Data for Steroids.

[12] Murashige T., Skoog F. (1962). A revised medium for rapid growth and bioassays with tobacco tissue culture. Physiol. Plant..

